# Photon-counting detector CT: a disrupting innovation in medical imaging

**DOI:** 10.1186/s41747-025-00571-4

**Published:** 2025-03-25

**Authors:** Akos Varga-Szemes, Tilman Emrich

**Affiliations:** 1 Department of Radiology and Radiological Science, Medical University of South Carolina, Charleston, SC USA; 2 Department of Diagnostic and Interventional Radiology, University Medical Center of the Johannes Gutenberg-University, Mainz, Germany; 3 German Centre for Cardiovascular Research, Partner site Rhine-Main, Mainz, Germany

**Keywords:** Computed tomography angiography, Disruptive technology, Photon-counting detector, Radiation dosage, Tomography (x-ray computed)

## Abstract

Over the past decades, computed tomography (CT) imaging has profited from various technical innovations. Besides improvements such as higher temporal and spatial resolutions, lower radiation dose, and the introduction of dual- and multi-energy imaging, the development and recent clinical introduction of photon-counting detector CT (PCD-CT) represents a milestone with the potential to substantially change clinical CT imaging and expand its indications. This thematic series of *European Radiology Experimental* comprises a collection of original research papers and review articles demonstrating the benefits and challenges of this cutting-edge technology. The thematic series includes a wide range of relevant topics spanning from initial clinical experiences using PCD-CT to original research papers covering potential applications in various body regions.

The field of medical imaging has witnessed several revolutionary advancements over the past decades. Some of these, such as the latest innovation, photon-counting detector computed tomography (PCD-CT), hold the promise to fundamentally transform clinical practice [1, 2]. PCD-CT represents a significant departure from conventional energy-integrating detector (EID) CT systems, which measure the total energy deposited by incoming x-rays without distinguishing between individual photons. EID-CT systems, therefore, have inherent limitations, such as limited spatial resolution and contrast-to-noise, especially at low radiation doses. In contrast, PCD-CT systems operate on a fundamentally different principle by counting individual x-ray photons and measuring the energy of each of them. This technological landscape paves the way for significant advancements in both experimental and clinical imaging, facilitating the transition of phantom studies into routine clinical applications. This sets the stage for the new thematic series in *European Radiology Experimental* titled “Photon-counting detector CT: a disrupting innovation in medical imaging,” which we are pleased to introduce with this editorial.

The disruptive potential of PCD-CT lies in its ability to not only address the limitations of EID-based systems but also to introduce new capabilities. By directly counting photons and categorizing them based on their energy, PCD-CT can produce images with higher spatial resolution, better contrast, and reduced noise [1]. Such improvements have the potential to dramatically enhance the diagnostic power of CT imaging across a wide range of clinical applications.

One of the most significant benefits of PCD-CT is its ability to improve image quality while simultaneously reducing radiation dose. By more accurately measuring the energy of incoming photons, PCDs can enhance tissue differentiation, yielding more detailed images in both spatial resolution and contrast resolution compared to conventional dual-energy EID-based systems, which primarily rely on superior spectral imaging capabilities.

The ability to perform energy-resolved imaging allows for the generation of various spectral-based reconstructions, including virtual mono-energetic images, iodine maps, virtual non-iodine, or virtual non-calcium images. This capability is especially valuable in detecting calcifications, distinguishing between types of tissue, and identifying contrast agents, all of which may be critical in accurate diagnosis and treatment planning. While dual-energy EID-CT systems can also deliver spectral information, the dual-source setup of the PCD-CT system with its temporal resolution of 66 ms (compared to 125 ms on a third-generation dual-source system in dual-energy mode) makes the use of spectral imaging feasible even for cardiac imaging. PCD-CT also reduces beam-hardening artifacts, which is achieved because PCDs can more effectively discriminate between low- and high-energy photons, minimizing the confounding effects of beam hardening [3, 4]. Another notable advantage is the potential for contrast media dose reduction. Using virtual mono-energetic image reconstructions, a 50% reduction in contrast media dose can still achieve diagnostic level attenuation for coronary CT angiography, as shown in a dynamic phantom study [5].

The unique benefits of PCD-CT make its use particularly advantageous in several clinical areas. Cardiovascular imaging, for instance, stands to benefit greatly from the enhanced spatial resolution (ultra-high resolution, *i.e*., 120 × 0.2 mm collimation) and spectral imaging capabilities of PCD-CT. The ability to quantify stenosis more accurately [6] and clearly visualize coronary artery lumen even in the presence of heavy calcification or stents [7] leads to a higher positive predictive value and a lower rate of unnecessary referrals for invasive testing (> 50% of patients reclassified to a lower risk category when using ultra-high resolution in a recent patient study), which ultimately result in the better management of patients with coronary artery disease [8].

Such benefits are also applicable to the various vascular structures across the body [9, 10]. PCD-CT may also be beneficial for the differentiation among various types of plaques, leading to earlier and more accurate characterization of coronary artery disease [11–13]. Finally, using PCD-CT, the ability to characterize the myocardium has also shown improvements [14, 15].

PCD-CT also holds promise in musculoskeletal imaging, especially for the imaging of large joints at ultra-high spatial resolution, for the quantification of gout deposits and the visualization of bone edema using its spectral capability, and for imaging of orthopedic implants due to its improved metal artifact reduction [16]. PCD-CT has been shown to deliver benefits for the accurate assessment if osseointegration of orthopedic joint replacement implants [17], and for the direct visualization of both cartilage and bone details of the knee using virtual mono-energetic images [18]. It also provides superior objective and subjective image quality and visualization for trabecular bone structure of the elbow and the radius, as well as for fracture depiction, compared to EID-CT [19, 20].

Without providing a comprehensive overview, all other areas of radiology have the potential to gain benefits from this new technology. The growing body of literature demonstrates the increasing research and clinical interest in PCD-CT, spanning from neuroradiology [21] through breast imaging [22] to a wide range of applications in pediatric imaging [23]. Latter especially benefits from the reduced radiation dose [24], combined with high-quality imaging, addressing the critical need for safe and effective imaging techniques in children, who are more sensitive to radiation and have a longer lifespan during which radiation-induced risks could manifest.

As we look into the future, the expansion of potential PCD-CT applications seems realistic. One area of ongoing research is the development of multicontrast imaging; *i.e*., a single scan imaging after the simultaneous injection of multiple contrast agents, such as iodine, gadolinium, or bismuth [25, 26]. Such multicontrast imaging, for example, can be used to visualize arterial and portal venous enhancement in a single scan to characterize liver lesions [27]. Further technical improvements, however, are expected to enable k-edge imaging tuned for non-iodine contrast agents [28].

Another area where we see the field is moving toward is the integration of PCD-CT with artificial intelligence (AI)-based applications. AI-driven deep learning-based noise reduction is one great example that can tackle the higher image noise especially observed in ultra-high-resolution images, which has been demonstrated in patients with multiple myeloma [29]. Beyond improving image quality, AI may also contribute to more granular quantitative image evaluation. PCD-CT-based radiomics, although not necessarily AI-based, however, often AI-powered, has been extensively investigated with the potential of identifying new imaging biomarkers in various conditions [30–32].

In the long run, as PCD-CT becomes more widely adopted, we may see the development of new clinical protocols and guidelines that take full advantage of the technology’s capabilities. This could lead to a shift in how certain conditions are diagnosed and treated, with PCD-CT playing a central role in the imaging toolbox. However, the widespread adoption of PCD-CT will also require addressing several challenges. These include the high cost of the technology, the need for specialized technologist training, and the development of standardized imaging protocols, which will most likely relevantly differ from conventional EID-CT protocols. As with any disruptive technology, there will be a period of adjustment as the medical community learns to fully integrate PCD-CT into clinical practice.

PCD-CT is more than just an incremental improvement over traditional EID-CT technology; it is a disruptive innovation that has the potential to transform CT imaging. Its ability to produce higher-quality images with lower radiation doses, reduce artifacts, and enhance tissue characterization makes it a powerful tool in the diagnosis and treatment of a wide range of conditions. The concept of “spectral CT” was first introduced by Godfrey N. Hounsfield, one of the two inventors of CT, in 1972 [33]. Now, more than 50 years later, we have only just begun to realize the full potential of this remarkable technology.

With this Editorial, we wish to deliver the invitation to the radiology community to contribute to this thematic series and expand our knowledge about PCD-CT.

## Abbreviations

AI: Artificial intelligence
CT: Computed tomography
EID: Energy-integrating detector
EID-CT: Energy-integrating detector computed tomography
PCD: Photon-counting detector computed tomography
